# The Stapled AKAP Disruptor Peptide STAD-2 Displays Antimalarial Activity through a PKA-Independent Mechanism

**DOI:** 10.1371/journal.pone.0129239

**Published:** 2015-05-26

**Authors:** Briana R. Flaherty, Yuxiao Wang, Edward C. Trope, Tienhuei G. Ho, Vasant Muralidharan, Eileen J. Kennedy, David S. Peterson

**Affiliations:** 1 Department of Infectious Diseases, University of Georgia College of Veterinary Medicine, Athens, Georgia, United States of America; 2 Center for Tropical and Emerging Global Diseases, University of Georgia, Athens, Georgia, United States of America; 3 Department of Pharmaceutical and Biomedical Sciences, College of Pharmacy, University of Georgia, Athens, Georgia, United States of America; 4 Department of Cellular Biology, University of Georgia, Athens, Georgia, United States of America; Food and Drug Administration, UNITED STATES

## Abstract

Drug resistance poses a significant threat to ongoing malaria control efforts. Coupled with lack of a malaria vaccine, there is an urgent need for the development of new antimalarials with novel mechanisms of action and low susceptibility to parasite drug resistance. Protein Kinase A (PKA) has been implicated as a critical regulator of pathogenesis in malaria. Therefore, we sought to investigate the effects of disrupted PKA signaling as a possible strategy for inhibition of parasite replication. Host PKA activity is partly regulated by a class of proteins called A Kinase Anchoring Proteins (AKAPs), and interaction between *Hs*PKA and AKAP can be inhibited by the stapled peptide Stapled AKAP Disruptor 2 (STAD-2). STAD-2 was tested for permeability to and activity against *Plasmodium falciparum* blood stage parasites *in vitro*. The compound was selectively permeable only to infected red blood cells (iRBC) and demonstrated rapid antiplasmodial activity, possibly via iRBC lysis (IC_50_ ≈ 1 μM). STAD-2 localized within the parasite almost immediately post-treatment but showed no evidence of direct association with PKA, indicating that STAD-2 acts via a PKA-independent mechanism. Furosemide-insensitive parasite permeability pathways in the iRBC were largely responsible for uptake of STAD-2. Further, peptide import was highly specific to STAD-2 as evidenced by low permeability of control stapled peptides. Selective uptake and antiplasmodial activity of STAD-2 provides important groundwork for the development of stapled peptides as potential antimalarials. Such peptides may also offer an alternative strategy for studying protein-protein interactions critical to parasite development and pathogenesis.

## Introduction

Malaria, caused by haemoprotozoan parasites of the genus *Plasmodium*, is endemic to nearly 100 countries, placing the lives of an estimated 3.2 billion people at risk each year. Despite widespread endemicity, intensified control efforts have reduced annual malaria-attributable deaths by approximately 47% from nearly 1 million in 2000 to 584 000 in 2013 [1]. Such advances in malaria control are critically dependent on effective disease diagnosis and efficacious drugs. Of the five species of *Plasmodium* currently known to infect humans, *P*. *falciparum* is the most pathogenic, accounting for the majority of malaria-related deaths, while *P*. *vivax* has a wider geographic distribution owing to its ability to survive in higher altitudes and cooler climates [1]. Since 2002, artemisinin combination therapy (ACT) has been the recommended first line treatment for uncomplicated *P*. *falciparum* malaria [2], and chloroquine is recommended for *P*. *vivax* in regions where it remains efficacious [1]. However, extensive resistance to all existing antimalarials, including growing resistance to artemisinin in the Greater Mekong Subregion, threatens to place control efforts, and millions of lives, in jeopardy [3–5].

As the threat of widespread artemisinin resistance looms, there is a growing need for antimalarials that are less vulnerable to parasite mechanisms of drug resistance. To date, all existing antimalarials, as well as most of those being pursued as potential candidates [6–10], are small molecule inhibitors. These drugs typically act by binding within tight, hydrophobic pockets of target proteins. Although many factors contribute to the development of drug resistant parasites, the binding restrictions of these small molecule inhibitors render them inherently vulnerable to loss of activity via random genetic mutations in the parasite. Most existing antimalarials have lost efficacy as a result of protein mutations that inhibit binding either to their target protein or to parasite transporters [11]. For example, mutations of residues within the binding pocket of the parasite’s dihydrofolate reductase led to resistance towards cycloguanil and pyrimethamine [12,13]; single mutations within the binding pocket of cytochrome b generated resistance to atovaquone [14–16]; mutations within the binding pockets of parasite transporters *Pf*CRT and *Pf*MDR1 eliminated activity of chloroquine and many ACT partner drugs (such as mefloquine and lumefantrine) [17,18]; and new evidence suggests mutations in the *Pf*Kelch13 propeller domain may be responsible for rising resistance to artemisinin [19–21]. As the search for the next antimalarial intensifies, there is an urgent need for new classes of inhibitors that act via unique mechanisms of action and possess reduced vulnerability to parasite drug resistance.

Stapled peptides are a novel class of inhibitors that can be designed to bind protein interfaces with high specificity and thereby block intra- or inter-molecular protein-protein interactions [22,23]. Unlike traditional protein therapeutics which are largely limited to extracellular targets, peptide stapling affords the ability to target a myriad of flat, elongated intracellular surfaces with high specificity due to its increased propensity for cell penetration [24]. Although many stapled peptides were originally engineered for various cancer targets [24,25], their unique potential as antimicrobial agents has recently been explored [26–28]. However, stapled peptides have not yet been tested in *Plasmodium*.

In the following study, we examined the permeability and activity of the stapled peptide, STAD-2, in *P*. *falciparum* infected red blood cells (iRBC) [29]. This peptide was originally designed to disrupt interaction between the regulatory subunits of human Protein Kinase A (PKA) and A Kinase Anchoring Proteins (AKAPs). PKA is a cAMP-dependent protein kinase that is critical for a wide variety of cellular processes. *Hs*PKA activity is highly regulated and dependent on multiple factors including intracellular cAMP concentrations and spatial and temporal localization via AKAP interactions [30–33]. AKAPs typically bind to the docking and dimerization domain (D/D) interface formed between two PKA regulatory (PKA-R) subunits. This docking site serves to recruit PKA to distinct subcellular locations and is a critical component of PKA regulation [29,30]. In *P*. *falciparum*, parasite PKA plays an important role in pathogenesis including regulation of protein phosphorylation, transport of molecules across the RBC membrane, and activation of “new permeability pathways” [34,35]. Further, RBCs release ATP in response to infection with *Plasmodium* parasites, which subsequently activates extracellular receptors to increase intracellular cAMP concentrations, thereby activating PKA. This signaling can ultimately cause deformations in the plasma membrane of both uninfected and infected RBCs [36]. While the role of AKAPs in healthy RBCs is poorly understood, recent work has shown that AKAPs play a critical role in RBC membrane stiffness and adhesion [37]. On the other hand, little is known regarding AKAPs in iRBCs; however, bioinformatics analyses have identified an ortholog of the *P*. *yoelii* AKAP within the *P*. *falciparum* genome [38]. In addition, subcellular localization of *Pf*PKA is thought to be critical for *Pf*PKA activity within iRBCs, suggesting a vital role for AKAPs in *P*. *falciparum* pathogenesis [39]. Much remains to be discovered regarding the roles of PKA and AKAPs in *P*. *falciparum* iRBCs as well as the interplay between parasite and host PKA in regulating PKA-dependent cellular processes.

Since many questions remain about the roles of *Hs*PKA and *Pf*PKA on *P*. *falciparum* pathogenesis and since the role of AKAPs is not well established in RBCs, we sought to explore the effects of treatment of *P*. *falciparum* iRBCs with the AKAP disruptor peptide STAD-2 (Stapled AKAP Disruptor 2). This work builds upon previous studies by Wang et al. which showed STAD-2 peptides were cell permeable in various mammalian cell lines and highly effective at inhibiting the intracellular interaction between *Hs*PKA-RII and AKAPs [29]. Although some important differences exist between *Pf*PKA and its human ortholog [38,40], *Pf*PKA was previously suggested as a promising antimalarial target [34,38]. Therefore, we sought to explore whether PKA could be exploited as a potential target for inhibiting parasite pathogenesis. STAD-2 was found to be selectively permeable to iRBC and, unexpectedly, demonstrated rapid antiplasmodial activity via a PKA-independent mechanism. Furosemide-insensitive, parasite-derived permeability appears to play a significant role in iRBC import of the compound. STAD-2 localized within the intracellular parasite but was not observed within the RBC cytosol. Further, STAD-2 did not clearly associate with the regulatory subunits of PKA, indicating STAD-2 likely acts through an alternative mechanism for inhibited parasite viability. This is the first example of uptake of a stapled peptide by *P*. *falciparum*-iRBCs, and these findings provide important groundwork for the development of stapled peptides for malaria-specific targets.

## Materials and Methods

### Blood and Reagents

Human O^+^ red blood cells were either purchased from Interstate Blood Bank, Inc. or donated by healthy volunteers. This research was approved by the Institutional Review Board (IRB) at the University of Georgia (no. 2013102100); all donors signed consent forms. Unless otherwise noted, all chemicals and reagents for this study were either purchased from Sigma Aldrich or Fisher Scientific.

### Parasite Culture and Synchronization

*Plasmodium falciparum* strains CS2, 3D7, Hb3, and Dd2 were maintained in continuous culture according to routine methods. Parasites were cultured at 4% hematocrit in O^+^ red blood cells. Cultures were maintained in 25 cm^2^ or 75 cm^2^ tissue culture flasks at 37°C under a gas mixture of 90% nitrogen/5% oxygen/5% carbon dioxide and in complete culture medium made up of RPMI containing 25 mM HEPES, 0.05 mg/mL hypoxanthine, 2.2 mg/mL NaHCO_3_ (J.T. Baker), 0.5% Albumax (Gibco), 2 g/L glucose, and 0.01 mg/mL gentamicin. Primarily ring-stage cultures were treated routinely with 5% D-Sorbitol to achieve synchronous cultures. Unless otherwise stated, experiments were carried out using the CS2 parasite strain.

### STAD-2 Synthesis and Purification

Peptides were synthesized and purified as previously described [29].

### STAD-2 Permeability

Synchronous ring-stage or late-stage infected red blood cells (iRBC) and uninfected red blood cells (uRBC) were brought up to 4% hematocrit in complete culture medium. FITC-conjugated peptides were added to a final concentration of 1 μM, and cultures were incubated for 6 hours at 37°C under standard gas conditions. Following incubation, 25 μL cell mixture was removed and treated with 100 μL 2 μg/mL Hoechst 33342 for 10 minutes at 37°C. Cells were subsequently washed once in 1 mL 1X PBS, resuspended in 300 μL 1X PBS, and analyzed for Hoechst and FITC staining on a Beckman Coulter CyAn flow cytometer. 500,000 events were collected at a rate of 15,000–20,000 events per second. Data was analyzed using FlowJo X single cell analysis software (FlowJo LLC).

### Dose-Response Curves

Synchronous ring-stage or late-stage iRBC at <0.5% parasitemia were brought up to 4% hematocrit in complete culture medium and transferred to a 24-well tissue culture plate in 1 mL aliquots. Wells were then brought up to final concentrations of 0.5, 1, 2, or 5 μM STAD-2 or STAD-2 scramble. 25 μL were removed from each well at 0, 24, 48, and 72 hours, stained with 100 μL 2 μg/mL Hoechst 33342 for 10 minutes at 37°C, and analyzed by flow cytometry as described above. IC_50_ values were determined according to parasitemia at 24 hours post-treatment. Parasitemia was defined as the percent of Hoechst-positive RBCs as measured by flow cytometry.

### Parasite Viability Assay

Ring-stage or late-stage iRBC at <0.5% parasitemia were brought up to 4% hematocrit in complete culture medium and transferred to a 24-well tissue culture plate in 1 mL aliquots. iRBCs were then treated with 1 μM STAD-2, 1 μM scrambled STAD-2, or 0.001% DMSO (vehicle control). 25 μL were removed from each well every 24 hours post-treatment, and samples were stained with Hoechst 33342 and analyzed by flow cytometry. Blood smears were made every 24 hours, fixed with methanol, and stained with Giemsa for analysis by light microscopy. Images were acquired using a Nikon Eclipse E400 microscope fitted with a Nikon Digital Sight DS-5M-L1 camera (Nikon Instruments Inc.).

### Hemolysis

Synchronous late-stage iRBC were mixed with uRBC in order to achieve a series of samples with stepwise decreasing parasitemia. Samples were brought up to 4% hematocrit in complete culture medium containing 1 μM STAD-2, 1 μM STAD-2 scramble, or 0.001% DMSO and transferred to a 48-well tissue culture plate in 200 μL aliquots in duplicate. The plate was incubated at 37°C under standard gas conditions for 6 hours. Following incubation, all samples were transferred to Eppendorf tubes and centrifuged at 700 rcf for 5 minutes to pellet cells. 100 μL of supernatant was removed from each tube and transferred to a 96-well, flat-bottom tissue culture plate. Oxyhemoglobin absorbance was measured at 415 nm using a SpectraMax Plus microplate spectrophotometer with SoftMax Pro 5.4 software (Molecular Devices, LLC).

### Fluorescence Microscopy

A culture of primarily late-stage 3D7 iRBC was brought up to 4% hematocrit in complete culture medium and transferred to a 24-well plate at 1 mL per well. FITC-conjugated STAD-2 or scrambled STAD-2 were added to a final concentration of 1 μM, and cultures were incubated for 10 minutes or 3 hours at 37°C under standard gas conditions. Following incubation, 50 μL of cell mixture was removed and immediately stained with 200 μL 2 μg/mL Hoechst 33342 for 10 minutes at 37°C. Cells were washed once with 1 mL 1X PBS, resuspended in 200 μL 4% paraformaldehyde/0.0075% glutaraldehyde, and deposited on coverslips pre-treated with 0.01% poly-L-lysine (Sigma Diagnostics) for 10 minutes. Post-fixation, coverslips were washed three times with 1X PBS, mounted on a glass microscope slide (Globe Scientific Inc.) with Fluoro-Gel anti-fading solution (Electron Microscopy Sciences), and sealed. Cells were imaged with a DeltaVision II microscope system using an Olympus IX-71 inverted microscope and a CoolSnap HQ2 CCD camera. Focal planes were selected based on transmitted light images. 0.2 μm z-stacks were acquired and deconvolved using SoftWorx 5.5 acquisition software (Applied Precision, Inc.).

### Immunofluorescence Assays

A culture of primarily late-stage iRBC was incubated with STAD-2 or DMSO as described above. Following incubation, 50 μL of cell mixture was removed and washed twice with 1 mL 1X PBS. Cells were then resuspended in 200 μL 4% paraformaldehyde/0.0075% glutaraldehyde and transferred to a CC2 glass chamber slide (Nalge Nunc International). Cells were fixed for 30 minutes at room temperature and gently washed twice with 300 μL 1X PBS. Fixed cells were subsequently permeabilized with 0.1% Triton X-100/PBS for 10 minutes and washed. Cells were then treated with 0.1 μg/mL sodium borohydride/PBS for 10 minutes to reduce any free aldehyde groups, washed with 1X PBS, and blocked for 1 hour at room temperature with 3% BSA/PBS. Following blocking, cells were brought up in 200 μL primary antibody and left shaking at 4°C overnight. The following morning, cells were washed three times, for 10 minutes each, to remove excess primary antibody and brought up in 200 μL secondary antibody for 1 hour at room temperature. After washing, cells were counterstained with 200 μL 0.5 μg/mL Hoechst 33342 for 3 minutes at room temperature and washed a final time before removing slide chambers, mounting a coverslip with Fluoro-Gel, and sealing. Rat anti-*Pf*PKA-R was generously donated by Gordon Langsley (Institut Cochin) and diluted 1:300 in 3% BSA/PBS. Goat anti-*Hs*PKA-RII (Abcam) was diluted to 10 μg/mL in 3% BSA/PBS. AlexFluor647 chicken anti-rat and AlexaFluor 647 donkey anti-goat secondary antibodies were diluted 1:500 in 3% BSA/PBS.

### Immunoprecipitation/Mass Spectrometry

4 X 10^8^ 3D7 parasites were lysed with cold 0.04% saponin in PBS for 10 minutes. The supernatant was, subsequently, separated from the pellet by centrifugation at 4000 rcf for 10 minutes at 4°C. This supernatant consisted of the RBC cytoplasm and parasite exported proteins (termed host supernatant). The pellet consisted of parasite cells; these were further lysed with cold 0.5% PBS. The soluble fraction (termed parasite supernatant) of the parasite cells was separated from the insoluble fraction by centrifuging the parasite lysate at 4000 rcf for 10 minutes at 4°C. The host supernatant and the parasite supernatant were each incubated with biotin-conjugated STAD-2 or STAD-2 scramble for 16 hours at 4°C. Peptide-protein complexes were precipitated using streptavidin-coupled Dynabeads (Life Technologies). Dynabeads were subsequently washed four times with 1X PBS, and the precipitated peptide-protein complexes were solubilized in SDS-PAGE sample buffer. Proteins were, then, fractionated on 10% SDS-PAGE, excised, and identified by MS-MS (Proteomics and Mass Spectrometry Core Facility, University of Georgia).

Sequences identified by MS-MS were searched against the NCBI non-redundant protein databases for *Plasmodium falciparum* (NCBI taxonomy #5833) and human (#9606)/*Plasmodium* (#5820) and analyzed by Mascot. Results from both database searches were cross-referenced and compiled. Proteins identified for STAD-2 and STAD-2 scramble treated lysates were compared and duplicates removed to yield a final list of STAD-2-specific protein interactions. Results are ordered by Mascot ions score (http://www.matrixscience.com/help/interpretation_help.html#SCORING). Generally, scores above 40 are considered possible interactions while scores above approximately 70 are considered good matches. All identified hits with a Mascot score above 40 are included in the supplementary tables.

### Coincubation Assays

Late-stage cultures were brought up to 4% hematocrit in complete culture medium and transferred to a 48-well tissue culture plate at 250 μL per well. Plates were spun at 330 rcf for 5 minutes, the medium was aspirated, and cultures were resuspended in either 250 μL complete culture medium or 200 μM furosemide in complete culture medium for 10 minutes at 37°C. Plates were again spun at 330 rcf, aspirated, and resuspended in 250 μL 1 μM STAD-2, 5% D-Sorbitol, 200 μM furosemide, 130 mM glycerol, 6 μM AgNO_3_, or a combination of the aforementioned for 10 minutes or 2 hours. Following incubation, 25 μL were removed, stained, and analyzed by flow cytometry as described above. Ten-minute time points served as controls for 5% D-Sorbitol and furosemide activities while effects on STAD-2 uptake were determined using 2-hour time points.

### Statistical Analysis

Graphing and statistical analyses were done using GraphPad Prism 5 (GraphPad Software, Inc.).

## Results

### STAD-2 is selectively permeable to infected red blood cells

**Fig 1:**
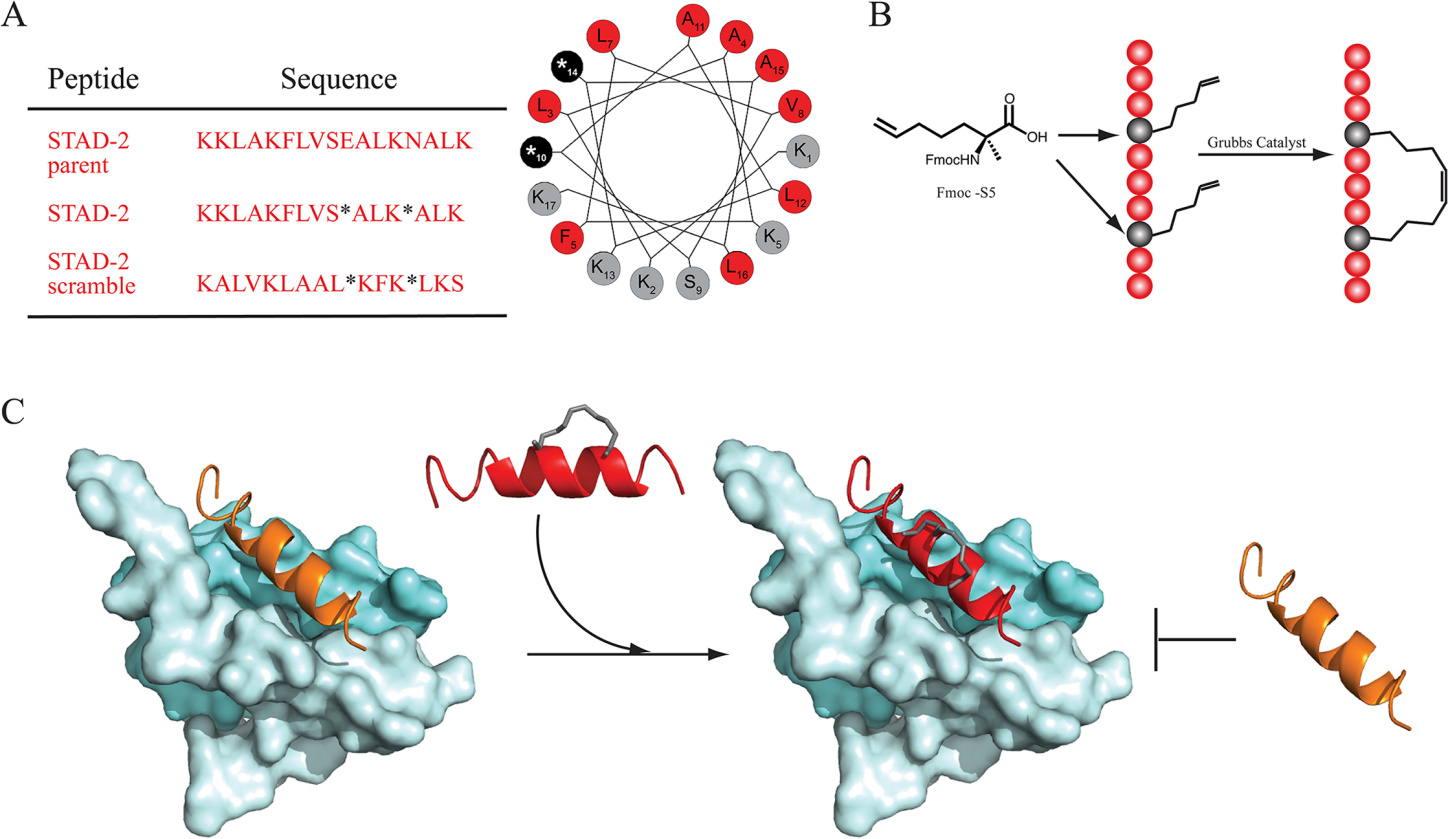
STAD-2 synthesis and function. (**A**) STAD-2 and STAD-2 scramble were synthesized by substituting S-pentenyl alanine (S_5_, shown as *) into positions that are opposite to the binding surface targeting PKA-R. A helical wheel represents the STAD-2 secondary structure wherein hydrophobic residues are shown in red, S_5_ in black, and other residues in grey. (**B**) Fmoc chemistry was used to synthesize STAD-2 peptides containing the non-natural S_5_ residues at *i*, *i*+4 positions. Ring-closing metathesis was performed using Grubbs I catalyst to generate the hydrocarbon staple. (**C**) The interaction between the *Hs*PKA D/D domain (pale cyan) and the docking sequence of an AKAP (orange) can be inhibited by the stapled disruptor peptide STAD-2 (red). STAD-2 mimics the docking sequence of an AKAP and disrupts binding to the regulatory subunit of PKA. Images were created using the crystal structure of PKA-RII (PDB access code: 2HWN [41]).

**Fig 2:**
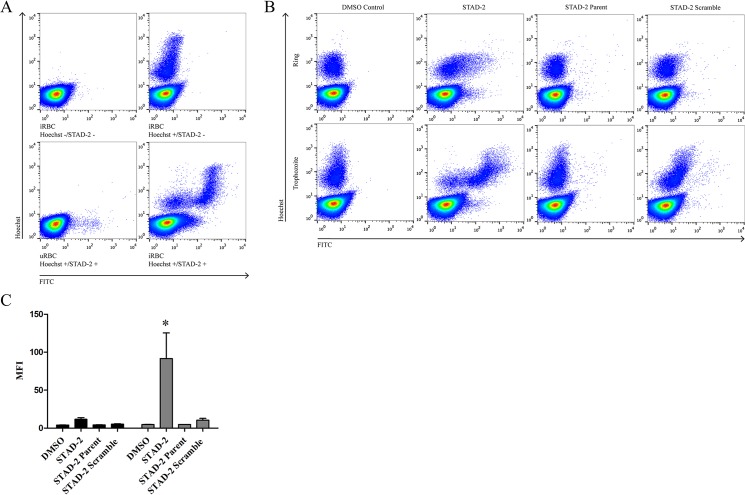
STAD-2 peptides are selectively permeable to *Plasmodium*-infected red blood cells. (**A**) *Plasmodium*-iRBC were treated for 6 hours with 1 μM FITC-conjugated STAD-2 and analyzed by flow cytometry. iRBC showed selective permeability to STAD-2 relative to uRBC. (**B, C**) Treatment of synchronous ring-stage (black bars) or late-stage (grey bars) cultures with 1 μM FITC-conjugated STAD-2, unstapled STAD-2 parent, or STAD-2 scramble demonstrated significantly increased uptake of STAD-2 by late-stage relative to ring-stage iRBC. However, both ring-stage and late-stage iRBC were minimally permeable to STAD-2 parent and STAD-2 scramble controls (2way ANOVA with Bonferroni posttest, p<0.001, n = 3–6, mean ± S.E.).

STAD-2 was designed to target the regulatory subunit of *Hs*PKA and occlude AKAP binding interactions as previously described [29] (Fig 1). To study the effects of STAD-2 in *Plasmodium*-infected red blood cells (iRBC), STAD-2 permeability was first examined by incubating 1 μM FITC-conjugated STAD-2 peptides with uninfected red blood cells (uRBC) and CS2 iRBC for 6 hours. Subsequent staining with 2 μg/mL Hoechst 33342 enabled separation of uninfected from infected red blood cells based on their DNA content. Analysis by flow cytometry showed significant permeability of STAD-2 in iRBC while only a nominal amount was permeable to uRBC (Fig 2A). Cell permeability patterns were consistent across other parasite strains tested (S1 Fig) and reached near-maximum levels by 3 hours post-treatment (S2 Fig).

*P*. *falciparum* has a blood stage life cycle of 44–48 hours wherein parasites upregulate new permeability pathways during the later stages of development. These pathways serve to facilitate uptake of solutes that are essential for parasite growth and division and are marked by the upregulation of a unique *Plasmodium* surface anion channel (PSAC) [42]. In order to examine the influence of parasite stage on uptake of STAD-2, iRBC were synchronized using 5% D-Sorbitol, and peptide permeability was measured in ring- and late-stage parasites. Late-stage iRBC demonstrated significantly increased uptake of STAD-2 relative to ring-stage parasites, consistent with the increased permeability of late-stage iRBC (Fig 2B and 2C). As controls, iRBC were also treated with the unstapled STAD-2 parent peptide as well as a scrambled version of STAD-2 possessing identical chemical composition but a scrambled amino acid sequence (Fig 1A). Permeability of these controls was almost negligible as measured by flow cytometry (Fig 2B and 2C), demonstrating selective uptake of STAD-2 by iRBC.

### STAD-2 reduces parasite viability

**Fig 3:**
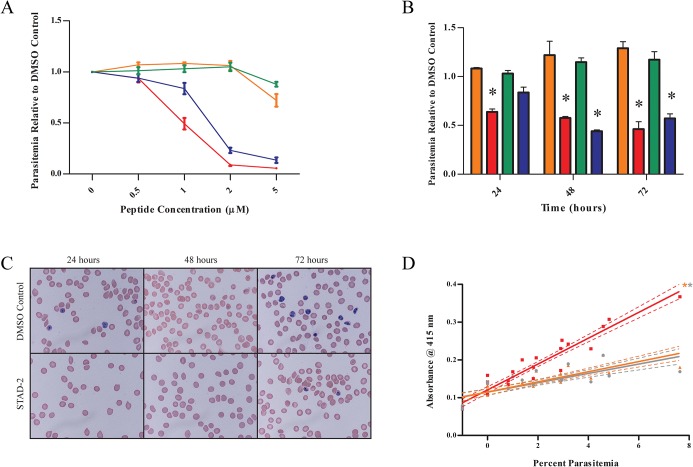
STAD-2 reduces viability of *P*. *falciparum in vitro*. (**A**) Synchronous ring- or late-stage iRBC were treated with 0, 0.5, 1, 2, or 5 μM STAD-2 or its scramble control, and parasitemia was determined by flow cytometry at 24-hours post-treatment. STAD-2 IC_50_ ≈ 1 μM for late-stage and 1.5 μM for ring-stage parasites (n = 3, mean ± S.E., red = STAD-2/late, blue = STAD-2/ring, orange = STAD-2 scramble/late, green = STAD-2 scramble/ring). (**B**) Synchronous iRBC were treated with 1 μM STAD-2 or STAD-2 scramble, and parasitemia was determined by flow cytometry at 24, 48 and 72 hours post-treatment. A significant decrease in parasitemia was seen with STAD-2 treatment of both ring- and late-stage iRBC (2way ANOVA, p<0.001, n = 3, mean ± S.E., red = STAD-2/late, blue = STAD-2/ring, orange = STAD-2 scramble/late, green = STAD-2 scramble/ring). (**C**) Analysis of cells from (**B**) by light microscopy showed STAD-2 treated iRBC to be morphologically indistinguishable from untreated controls. (**D**) Late-stage iRBC of increasing parasitemia were treated with 1 μM STAD-2, 1 μM STAD-2 scramble, or DMSO control for 6 hours. Since the presence of oxyhemoglobin is indicative of red blood cell lysis, culture medium was removed and analyzed for evidence of oxyhemoglobin (A_415_) by UV-Vis spectroscopy. Linear regression demonstrates positive correlation of cell lysis with increasing parasitemia and significantly increased lysis in STAD-2 treated cells relative to STAD-2 scramble and DMSO controls (p<0.0001, n = 4).

Since STAD-2 was selectively permeable to iRBC, we wanted to determine whether STAD-2 treatment had an effect on parasite viability *in vitro*. We first treated ring- and late-stage iRBC with 0, 0.5, 1, 2, and 5 μM STAD-2 or STAD-2 scramble for 24 hours and subsequently determined parasitemia relative to DMSO-treated controls. While treatment with the scramble control had negligible effects on parasite viability at all concentrations tested, a concentration-dependent decrease in parasitemia was observed in STAD-2-treated iRBC 24 hour post-treatment (Fig 3A). Consistent with increased STAD-2 permeability in late-stage iRBC (Fig 2B and 2C), late-stage parasites were slightly more susceptible to STAD-2 inhibition (IC_50_ ≈ 1 μM) than ring-stage parasites (IC_50_ ≈ 1.5 μM).

To further explore the effects of STAD-2 on parasite viability *in vitro*, synchronous ring- and late-stage iRBC were treated with 1 μM STAD-2 or scrambled STAD-2, and parasitemia was determined by flow cytometry in conjunction with light microscopy every 24 hours for 72 hours post-treatment. Again, STAD-2 scramble had no effect on parasite viability whereas both late- and ring-stage iRBC had significantly decreased parasitemias relative to their DMSO controls by 48 hours post-STAD-2 treatment (Fig 3B, p<0.001). STAD-2, likewise, inhibited several other parasite strains tested (S3 Fig). Interestingly, the morphology of STAD-2-treated iRBC did not differ from DMSO controls (Fig 3B).

Throughout the previous experiments, it was noticed that parasitemia was decreased as early as 6 hours post-treatment as detected by flow cytometry (S4 Fig). This, combined with the lack of morphological effects following STAD-2 treatment (Fig 3B), led us to explore whether STAD-2 was inducing lysis of iRBC. To test this hypothesis, we prepared serial dilutions of late-stage iRBC in uRBC in order to achieve a series of samples ranging from 0 to 8% parasitemia. We then treated the samples with 1 μM STAD-2 or STAD-2 scramble for 6 hours and quantified cell lysis by measuring the absorbance of oxyhemoglobin in the sample medium (A_415_). While levels of lysis induced by STAD-2 scramble were essentially identical to DMSO controls, STAD-2-induced lysis was significantly greater than controls and directly correlated with increasing parasitemia (Fig 3D). Hence, we propose that STAD-2-induced cell lysis is specific to *P*. *falciparum* infected cells.

### STAD-2 traffics to the intracellular parasite

**Fig 4:**
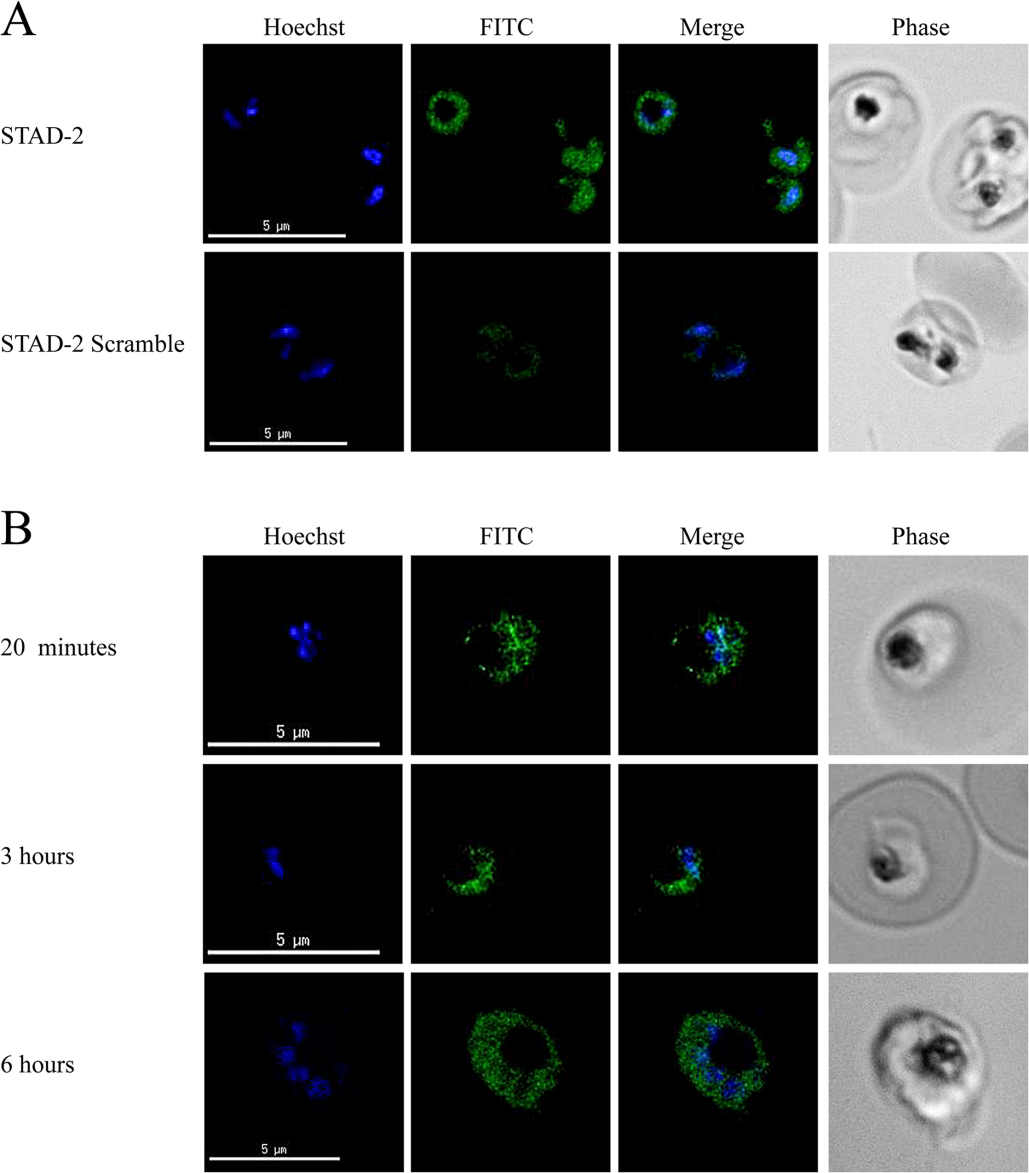
STAD-2 rapidly localizes within the parasitophorous vacuole. (**A**) 3D7 iRBC were treated with 1 μM STAD-2 or STAD-2 scramble, stained with 2 μg/mL Hoechst 33342, and analyzed by fluorescence microscopy. STAD-2 peptides consistently localized within the intracellular parasite and at much higher levels than its scrambled control. (**B**) iRBC treated for 20 minutes, 3 hours, or 6 hours with 1 μM STAD-2 showed that STAD-2 traffics to the parasitophorous vacuole by 20 minutes post-treatment.

Currently, there is nothing known concerning AKAPs in relation to *P*. *falciparum* iRBC. Thus, we wanted to probe whether STAD-2 localized within the host red blood cell or within the parasite. In order to determine intracellular localization of FITC-conjugated STAD-2, iRBC were treated and stained as before and analyzed by fluorescence microscopy. Repeated analyses found that STAD-2 consistently localized within the parasite but rarely within the parasite digestive vacuole (Fig 4A). At no point was STAD-2 observed within the red blood cell cytosol. This pattern of peptide localization was consistent in both fixed and unfixed cells as well as both undivided (trophozoite) and divided (schizont) late-stage parasites. In addition, we found that STAD-2 quickly reached intracellular parasites, localizing within parasites as early as 20 minutes post-treatment, and maintained an identical pattern of localization as late as 6 hours post-treatment (Fig 4B.). Thus, selective uptake and localization of STAD-2 seems independent of any changes in parasite morphology during late-stage development.

### STAD-2 does not associate with PKA

**Fig 5:**
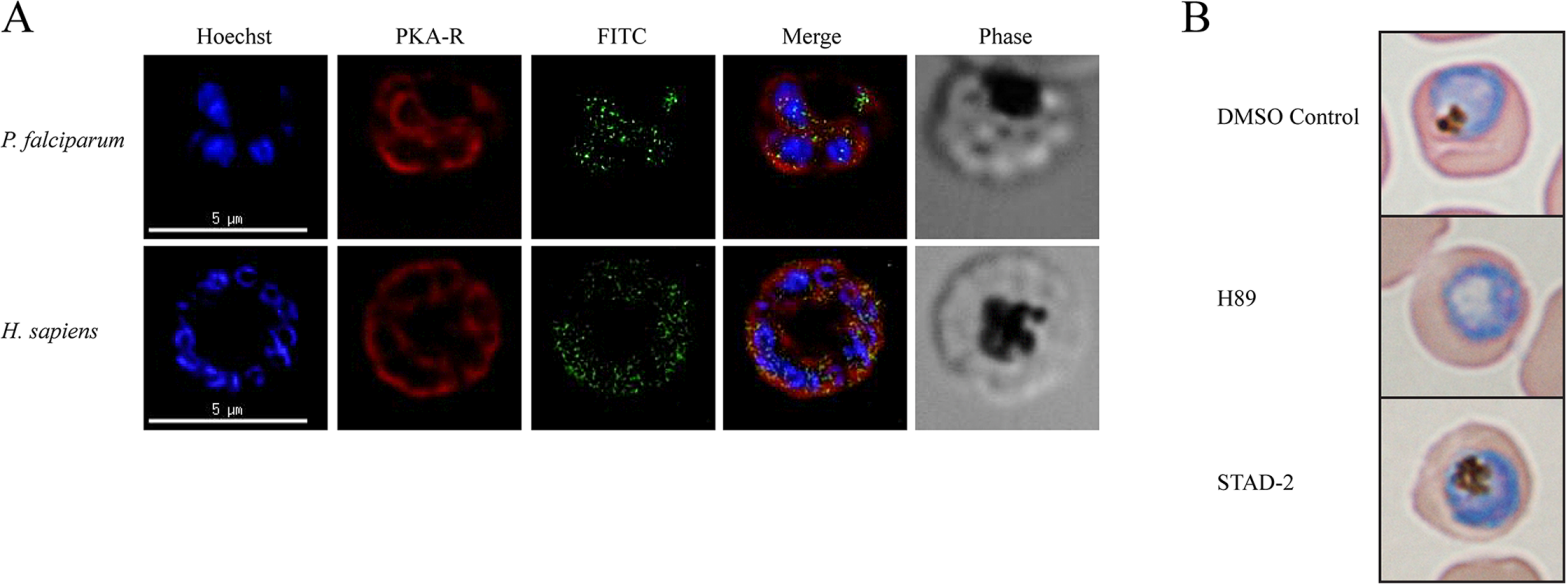
STAD-2 does not associate with PKA. (**A**) Late-stage iRBC were treated with 1 μM FITC-conjugated STAD-2 for 2 hours and probed for *P*. *falciparum* PKA-R (top panel) or *H*. *sapiens* PKA-RII (bottom panel). STAD-2 did not show clear colocalization with either of the regulatory subunits. (**B**) Late-stage iRBC were treated with 1 μM STAD-2, 30 μM H89 (small molecule inhibitor of PKA), or 0.001% DMSO and analyzed by light microscopy at 48 hours post-treatment. H89-treated iRBC demonstrated clear absence of parasite digestive vacuoles while STAD-2 treated iRBC were indistinguishable from DMSO controls.

Since STAD-2 localizes within the parasite and not within the cytoplasm of the red blood cell, we wanted to determine if STAD-2 targets PKA within the parasite. We first addressed this question using immunofluorescence assays of iRBC treated with FITC-conjugated STAD-2 and probed with either anti-*Pf*PKA R or anti-*Hs*PKA RII antibodies. STAD-2 did not definitively colocalize with either of the regulatory subunits tested in iRBC (Fig 5A), although lack of colocalization with *Hs*PKA can likely be attributed to the compound’s inability to accumulate in the RBC cytoplasm. Likewise, coimmunoprecipitation experiments using biotin-conjugated STAD-2 did not yield detectable levels of the probed PKA subunits by western blotting (data not shown) nor were they evident as interacting partners when analyzed by mass spectrometry (S1 Table and S2 Table). Finally, we compared the effects of treatment with STAD-2 to those of the PKA small molecule inhibitor, H89. Both STAD-2 and H89 were applied at their relative IC_50_ values (1 μM and 30 μM, S5 Fig) and analyzed by blood smears at 48 hours post-treatment. Although STAD-2-treated iRBC did not differ morphologically from DMSO controls, H89-treated iRBC consistently showed clear morphological changes including the notable absence of a digestive vacuole (Fig 5B). Thus, we suggest that STAD-2 may act via an alternate mechanism that does not involve disruption of PKA signaling.

### STAD-2 is internalized via unknown parasite permeability pathways

**Fig 6:**
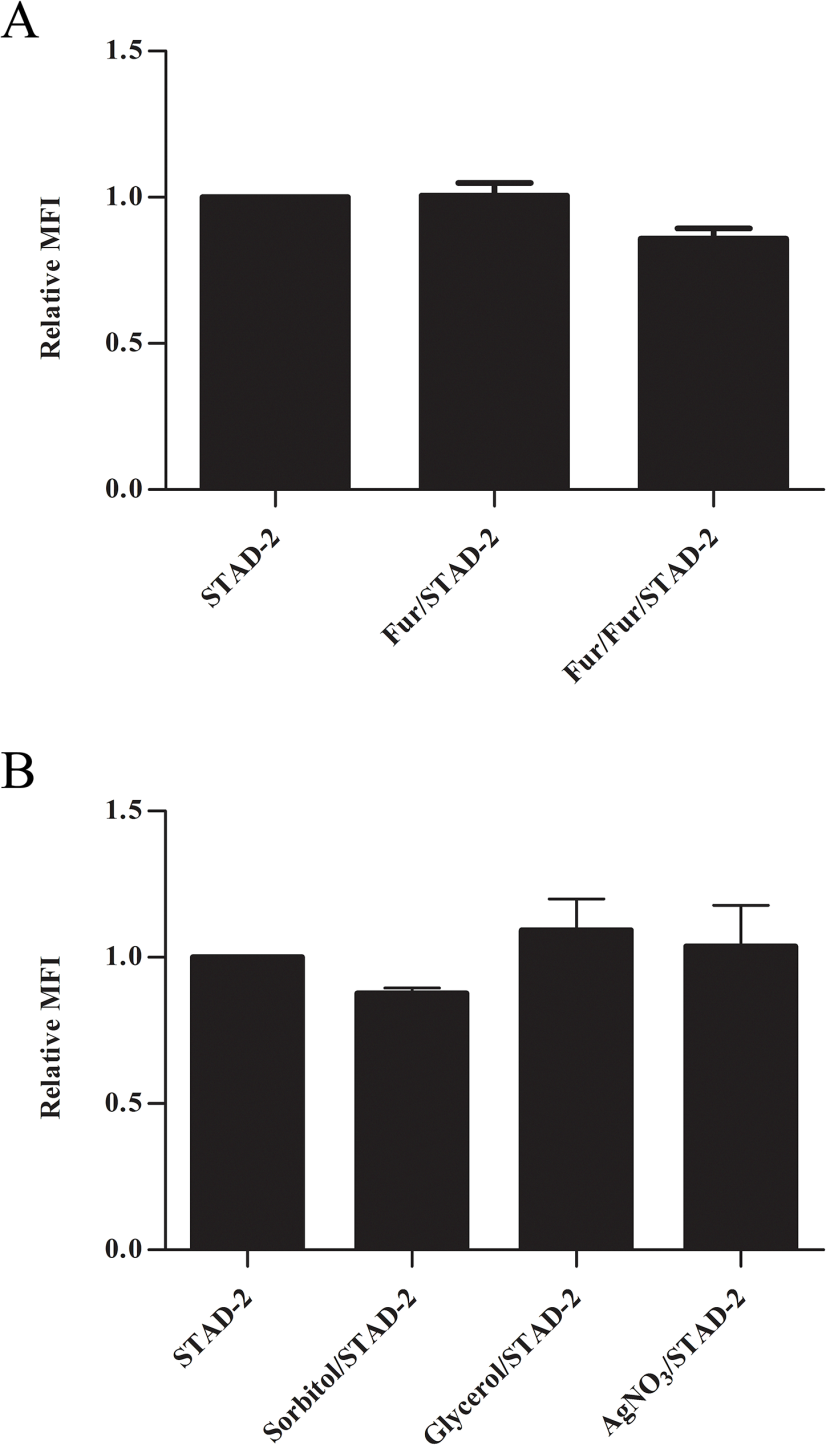
STAD-2 uptake is largely independent of the PSAC. (**A**) Late-stage iRBC were treated with 1 μM STAD-2 in the presence of 200 μM furosemide following pre-treatment with complete culture medium (Fur/STAD-2) or 200 μM furosemide (Fur/Fur/STAD-2). Treatment of late-stage iRBC with STAD-2 in the presence of furosemide demonstrated a visible, yet insignificant, decrease in STAD-2 uptake only when iRBC were pre-treated with furosemide (two-tailed t test, p = 0.0557, n = 3, mean ± S.E.). (**B**) Late-stage iRBC were treated with 1 μM STAD-2 in the presence of 5% D-Sorbitol (PSAC solute), 130 mM glycerol (AQP3 solute), or 6 μM AgNO_3_ (AQP1 inhibitor). Treatment with STAD-2 in the presence of 5% D-Sorbitol yielded a decrease in STAD-2 uptake similar to that seen in (**A**) while treatment in the presence of glycerol or AgNO_3_ did not differ from STAD-2 alone (n = 2, mean ± S.E.).

Although it is well established that *Plasmodium* parasites have increased permeability to extracellular solutes during the latter half of the blood-stage life cycle, many questions remain regarding the mechanisms of permeability. It is generally accepted that smaller solutes are imported via a PSAC that is upregulated by the parasite throughout the course of development; however considerably less is understood regarding uptake of larger solutes. In order to better understand how the relatively large ~2.5 kDa peptide, STAD-2, gains intracellular access to iRBC, we measured uptake of 1 μM STAD-2 in the presence of the PSAC inhibitor furosemide. Although STAD-2/furosemide co-treatment alone had no discernible effect on STAD-2 uptake, pre-incubation of iRBC with 200 μM furosemide followed by a 2-hour co-treatment showed a consistent but statistically insignificant decrease in STAD-2 uptake (Fig 6A). Thus, it appears that STAD-2 import does not heavily rely on the PSAC.

Recent studies have shown that some AKAPs associate with membrane aquaporins and play a role in aquaporin phosphorylation and channel regulation within various cell types [43,44]. Although evidence suggests the presence of both aquaporin 1 (AQP1) and aquaporin 3 (AQP3) on the surface of healthy red blood cells [45], few studies have examined the role of membrane aquaporins in *P*. *falciparum* iRBC. However, AQP3 is thought to be the major glycerol channel in human erythrocytes and may play a role in the virulence of intraerythrocytic parasites [46]. To further explore the role of membrane transport mechanisms in STAD-2 uptake, we performed co-incubation experiments similar to those above using D-Sorbitol (PSAC solute), glycerol (AQP3 solute [47,48]), or AgNO_3_ (AQP1 inhibitor [49]). Co-incubation of iRBC with 1 μM STAD-2 and 130 mM glycerol or 6 μM AgNO_3_ yielded no apparent change in STAD-2 uptake; however, co-incubation with 5% D-Sorbitol resulted in a slight reduction of STAD-2 uptake that was comparable to that seen with furosemide (Fig 6B). Thus, although AQP1 and AQP3 are unlikely to play a role in STAD-2 uptake or activity, the PSAC may be responsible for a fraction of STAD-2 import. Nevertheless, we propose that none of the assessed means of transport greatly contribute to the uptake of STAD-2 by iRBC.

### STAD-2 permeability is peptide-specific

**Fig 7:**
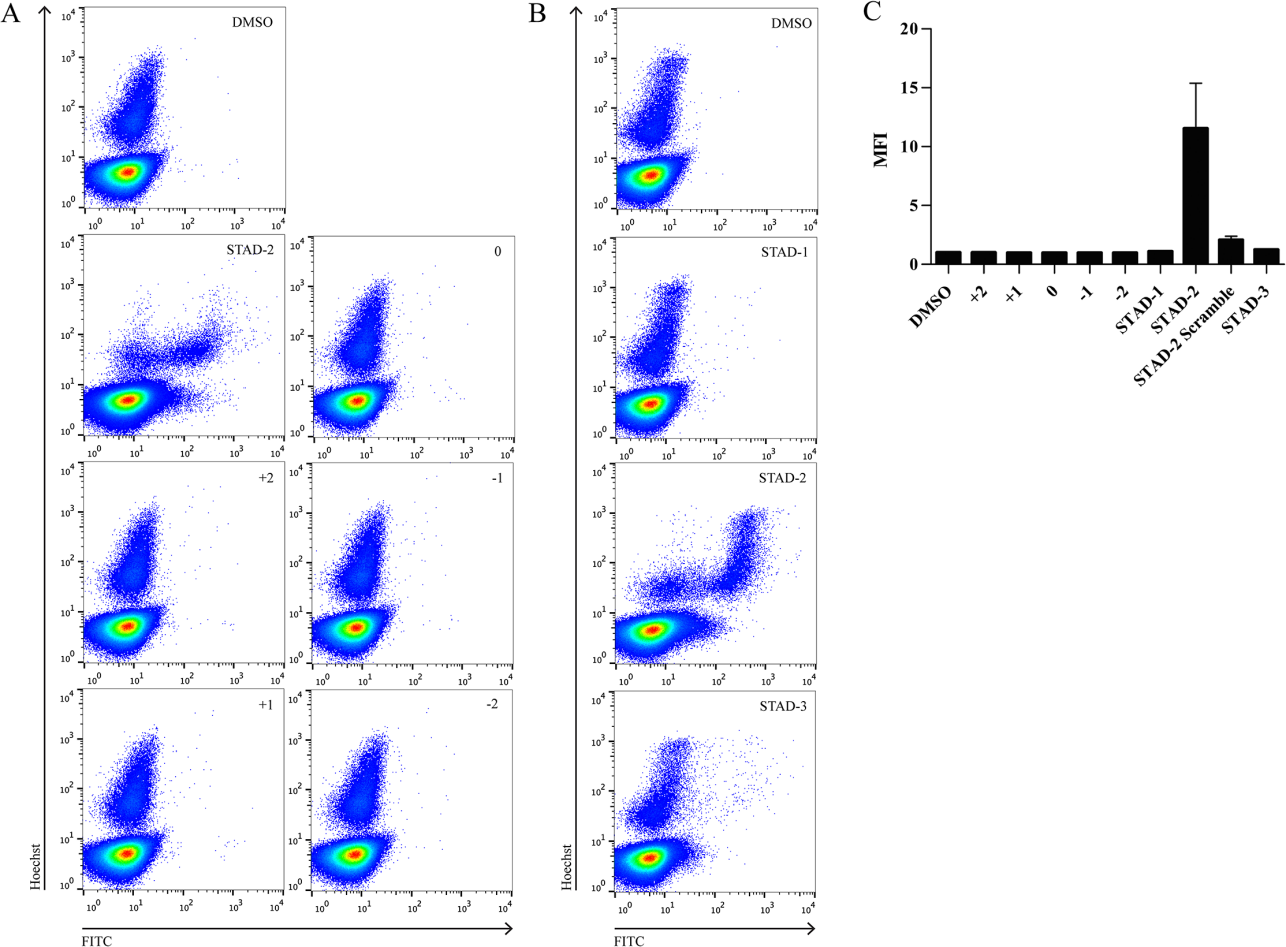
STAD-2 is uniquely permeable to iRBC. iRBC were treated for 6 hours with 1 μM stapled peptides of varying charges (**A**) or variants of STAD-2 (**B**), analyzed by flow cytometry, and reported as median fluorescence intensity (**C**, n = 2–6, mean ± S.E.). Of the various stapled peptides analyzed, only STAD-2 was clearly permeable to iRBC.

Since none of the examined membrane channels contributed substantially to the uptake of STAD-2, we further explored the requirements of iRBC permeability by testing for uptake of a variety of different stapled, FITC-conjugated peptides. All peptides were applied at a concentration of 1 μM for 6 hours and analyzed by flow cytometry as previously described. The overall net charge and amino acid sequence of all peptides tested are shown in Table 1. The first series of peptides analyzed explored the influence of peptide charge on iRBC uptake (Fig 7A) while the second series examined uptake of other STAD peptides that were developed previously (Fig 7B) [29]. Unexpectedly, STAD-2 was the only peptide that demonstrated clear permeability to iRBC (Fig 7C) providing further evidence that iRBC uptake of STAD-2 is highly specific.

**Table 1:** Stapled Peptides.

Peptide	Sequence	Charge	Length*
+2 Charged	Beta-Ala **S**_**5**_ K K L **S**_**5**_ T T	+2	8
+1 Charged	Beta-Ala **S**_**5**_ K G L **S**_**5**_ T T	+1	8
Neutral Charged	Beta-Ala **S**_**5**_ G G L **S**_**5**_ T T	0	8
-1 Charged	Beta-Ala **S**_**5**_ E G L **S**_**5**_ T T	-1	8
-2 Charged	Beta-Ala **S**_**5**_ E E L **S**_**5**_ T T	-2	8
STAD-1	Beta-Ala K K Y A K Q L A D **S**_**5**_ I I K **S**_**5**_ A T E	+2	18
STAD-2	Beta-Ala K K L A K F L V S **S**_**5**_ A L K **S**_**5**_ A L K	+5	18
STAD-2 Scramble	Beta-Ala K A L V K L A A L **S**_**5**_ K F K **S**_**5**_ L K S	+5	18
STAD-3	Beta-Ala K K Y A Q R L S K K I V R A V **S**_**5**_ Q W A **S**_**5**_	+6	21

*Length in amino acids

## Discussion

With *Plasmodium* drug resistance on the rise, malaria control efforts are in desperate need of new antimalarials that are highly efficacious and largely refractory to parasite mechanisms of drug resistance. For the time being, artemisinin combination therapy (ACT) remains the WHO-recommended first line treatment for *P*. *falciparum* malaria. And, although resistance in the Greater Mekong Subregion has hampered the efficacy of artemisinin monotherapies, ACT currently remains highly effective at curing malaria provided it is paired with an efficacious partner drug [50]. However, mounting evidence of delayed clearance times places even ACTs in jeopardy and further underscores the need for new antimalarials [51].

In 2009, a high throughput phenotypic screen of nearly 2 million compounds from GlaxoSmithKline identified 13,533 compounds that inhibited growth of *P*. *falciparum* by greater than 80% at 2 μM concentration [52]. Of the compounds identified and validated in the study, a large majority were protein kinase inhibitors. Although *P*. *falciparum* has a relatively small kinome, composed of less than 100 identified kinases, many are highly divergent from those within the human host [40,53]. Furthermore, since many kinase signaling pathways are evolutionarily conserved, it has been suggested that a single kinase inhibitor might exhibit pluripotency and, therefore, reduced vulnerability to drug resistant mutations [53]. Thus, protein kinases represent promising candidates as future antimalarial targets.

In the present study, we explored the effects of the human AKAP disruptor peptide STAD-2 on *P*. *falciparum* iRBC. We demonstrated STAD-2 to be highly and selectively permeable to iRBC. In addition, STAD-2 effectively inhibited parasite viability at IC_50_ ≈ 1 μM. Surprisingly, analyses using antibodies specific to both *P*. *falciparum* and *H*. *sapiens* PKA regulatory subunits found no evidence of association of STAD-2 with human or parasite PKA within the iRBC. Likewise, parasite responses to treatment with the PKA small molecule inhibitor H89 differed considerably from treatment with STAD-2, providing further evidence that STAD-2 may not inhibit PKA regulation in iRBC. This is most likely due to the fact that STAD-2 does not accumulate in the RBC cytosol, yielding the human PKA-R target inaccessible, and *Pf*PKA-R does not contain the conserved D/D domain that is targeted by STAD-2. However, since *Hs*PKA-R is known to play a critical role in membrane stiffness and adhesion [37], it will be interesting to study the role of human AKAPs on *Plasmodium*-iRBCs through selective delivery of STAD-2 into the RBC cytoplasm. In an effort to dissect the mechanism of iRBC import of STAD-2, we found that intake was largely independent of the PSAC. In addition, import was highly sequence-specific since other stapled peptides, even some bearing very similar composition to STAD-2, were largely impermeable to iRBC.

This research provides the first support for the use of stapled peptides as potential candidates for exploring malaria-specific signaling and inhibition in RBCs. Although further study will be necessary to dissect the highly specific mechanisms of uptake seen with STAD-2, the selective permeability of stapled peptides to *P*. *falciparum* iRBC may ensure low cytotoxicity of future antimalarial stapled peptides.

As experiments examining the mechanism of peptide uptake did not show significant contributions by neither the PSAC nor resident RBC aquaporins, it remains unclear exactly how STAD-2 is imported into the parasite. Aside from the PSAC, permeability of iRBC is poorly understood. Various studies have shown high permeability of positively charged cell-penetrating peptides in *P*. *falciparum*-iRBC. These peptides typically possess octaarginine or octalysine structures and are thought to be taken up by endocytic mechanisms [54–56]. Also, like STAD-2, cell-penetrating peptides demonstrate selective permeability to iRBC relative to healthy uRBC [57]. However, the lack of permeability of control positively charged peptides in this study, particularly STAD-2 scramble and STAD-3, suggests that there may be more at play here than non-selective endocytosis. Therefore, it is possible that STAD-2 may be imported through a specific protein interaction that dictates permeability.

Although efforts to identify the mechanism of action of STAD-2 peptides within iRBC were inconclusive, it is interesting to consider the observed lytic activity of STAD-2 within iRBC. This activity may have important implications regarding STAD-2 function in *P*. *falciparum*. Previous studies have demonstrated a role for *Pf*PKA in regulation of parasite permeability such that overexpression of *Pf*PKA-R led to inhibition of anionic channel conductance [35]. In addition, characterization of *Pf*PKA-C from iRBC found highest activity of *Pf*PKA-C in schizont-infected cells, consistent with our observed STAD-2 activity in late-stage parasites [58]. Therefore, although we found no definitive association of STAD-2 with PKA-R, it is possible that STAD-2-induced iRBC lysis results from altered activity of PKA. Alternatively, our IP/MS experiments showed association of STAD-2 with cytoadherence linked asexual protein 3.1, or CLAG3, in the parasite supernatant (S2 Table). Recent studies have shown CLAG3 to be associated with the PSAC such that CLAG3 alters erythrocyte permeability [59,60], raising the possibility that STAD-2 may interfere with CLAG3 function and/or PSAC activity, thereby leading to iRBC lysis.

## Conclusions

Parasite drug resistance poses a serious threat to ongoing malaria control and elimination efforts. Development of new antimalarials targeting essential parasite components, such as kinases, or utilizing novel mechanisms of action may prevent devastating losses in our advancement toward malaria elimination. Here, we provide important groundwork for the use of stapled peptides as a novel class of antimalarials. Our data demonstrate the stapled AKAP disruptor, STAD-2, to be selectively permeable to iRBC. Furthermore, iRBC uptake of STAD-2 is highly specific and not reliant on known parasite permeability pathways. Notably, iRBC lysis triggered by STAD-2 appears to occur through a yet unknown, PKA-independent mechanism. Future research will explore the efficacy of stapled peptides designed to specifically inhibit protein-protein interactions unique to kinase targets in *P*. *falciparum*. We believe that the high specificity and unique mechanism of action of stapled peptides may yield promising new antimalarials with reduced vulnerability to parasite drug resistance.

## Supporting Information

S1 Fig STAD-2 is permeable to *P. falciparum* strains *in vitro*. Late-stage iRBC were treated with 1 μM FITC-conjugated STAD-2 for 2 hours, stained with 2 μg/mL Hoechst 33342, and analyzed for STAD-2 uptake by flow cytometry. CS2, Dd2, 3D7, and Hb3 parasite strains demonstrate comparable levels of permeability to STAD-2 peptides (n = 2).
(TIF)

S2 Fig STAD-2 permeability increases with time. Late-stage iRBC were treated with 1 μM FITC-conjugated STAD-2 for 1, 2, 3, or 6 hours and subsequently stained with 2 μg/mL Hoechst 33342 before analysis by flow cytometry. Near-maximum levels of STAD-2 uptake are evident by 3 hours post-treatment (n = 3, mean ± S.E.).
(TIF)

S3 Fig STAD-2 reduces viability of *P*. *falciparum* strains *in vitro*. Late-stage CS2, 3D7, Dd2, and Hb3 parasite strains were treated with 1 μM FITC-conjugated STAD-2, and parasitemia was determined by flow cytometry at 24, 48 and 72 hours post-treatment. STAD-2 demonstrated variable antimalarial activity between strains and reduced viability in CS2, Dd2, and Hb3 strains (n = 3, mean ± S.E.).
(TIF)

S4 Fig STAD-2 reduces parasitemia 6 hours post-treatment. Late-stage iRBC were treated with 1 μM FITC-conjugated STAD-2 for 6 hours, stained with 2 μg/mL Hoechst 33342, and analyzed by flow cytometry. Reduction in parasitemia was evident as early as 6 hour post-treatment (n = 7, mean ± S.E.).
(TIF)

S5 Fig H89 Dose-Response Curve. Late-stage iRBC were treated with serial dilutions (100, 50, 25, 12.5, 6.24, 3.13, 1.56, 0.78, and 0 μM) of the small molecule PKA inhibitor, H89, for 24 hours and stained with 2 μg/mL Hoechst 33342 for parasitemia analysis by flow cytometry. H89 *in vitro* IC_50_ ≈ 30 μM in late-stage CS2 parasites (n = 2, mean ± S.E.).
(TIF)

S1 Table Top hits from Host Supernatant. Identified STAD-2 interactors from host supernatant following IP and LC-MS/MS. Interactors are ranked based upon their Mascot ions score.
(TIF)

S2 Table Top hits from Parasite Supernatant. Identified STAD-2 interactors from parasite supernatant following IP and LC-MS/MS. Interactors are ranked based upon their Mascot ions score.
(TIF)
