# Supplementary figures and images for: The Stapled AKAP Disruptor Peptide STAD-2 Displays Antimalarial Activity through a PKA-Independent Mechanism
Source: PLoS One. 2015 May 26;10(5):e0129239. doi: 10.1371/journal.pone.0129239 (PMC4444124; doi:10.1371/journal.pone.0129239)

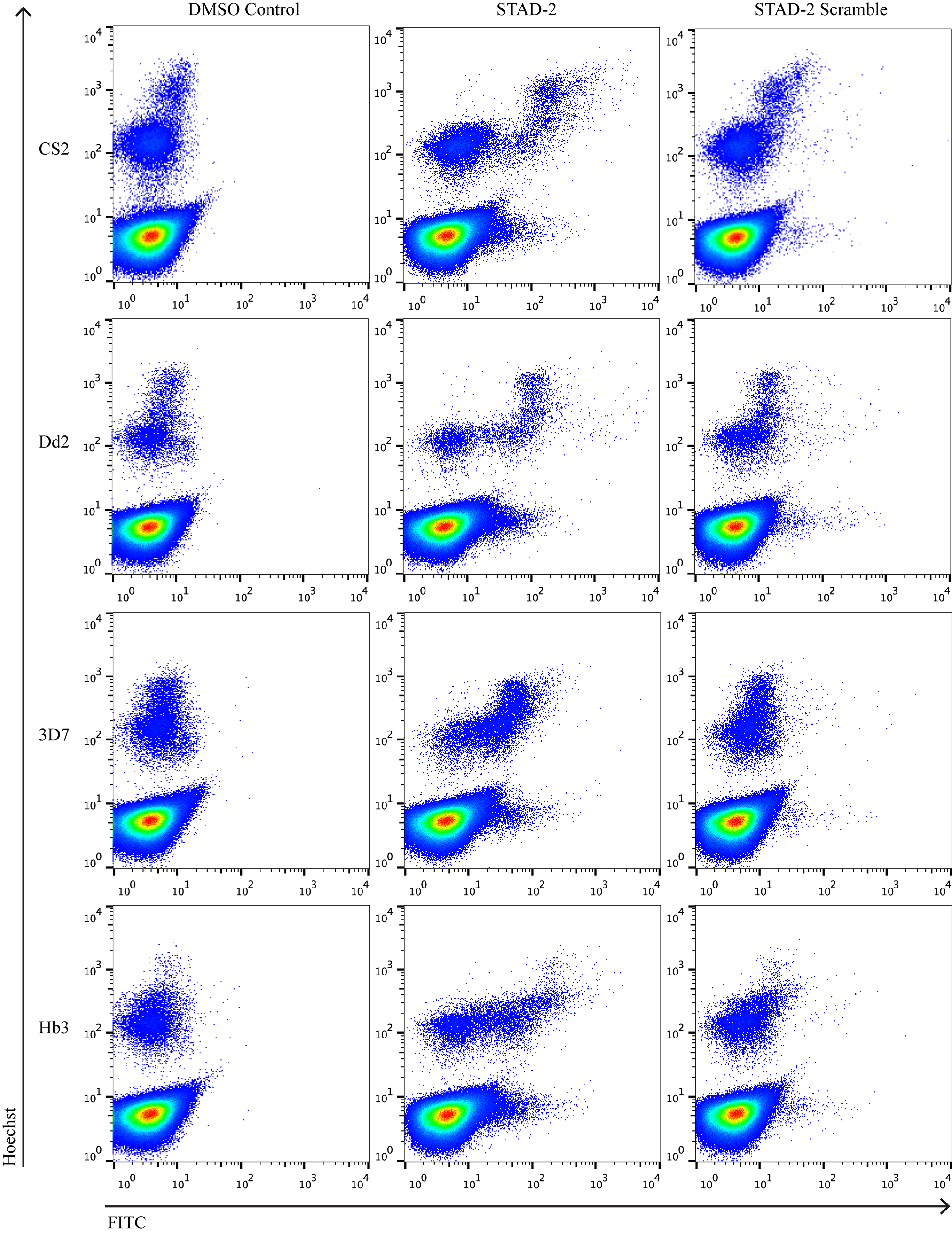

Supplement: S1 Fig — Late-stage iRBC were treated with 1 μM FITC-conjugated STAD-2 for 2 hours, stained with 2 μg/mL Hoechst 33342, and analyzed for STAD-2 uptake by flow cytometry. CS2, Dd2, 3D7, and Hb3 parasite strains demonstrate comparable levels of permeability to STAD-2 peptides (n = 2). (TIF) [file pone.0129239.s001.tif]

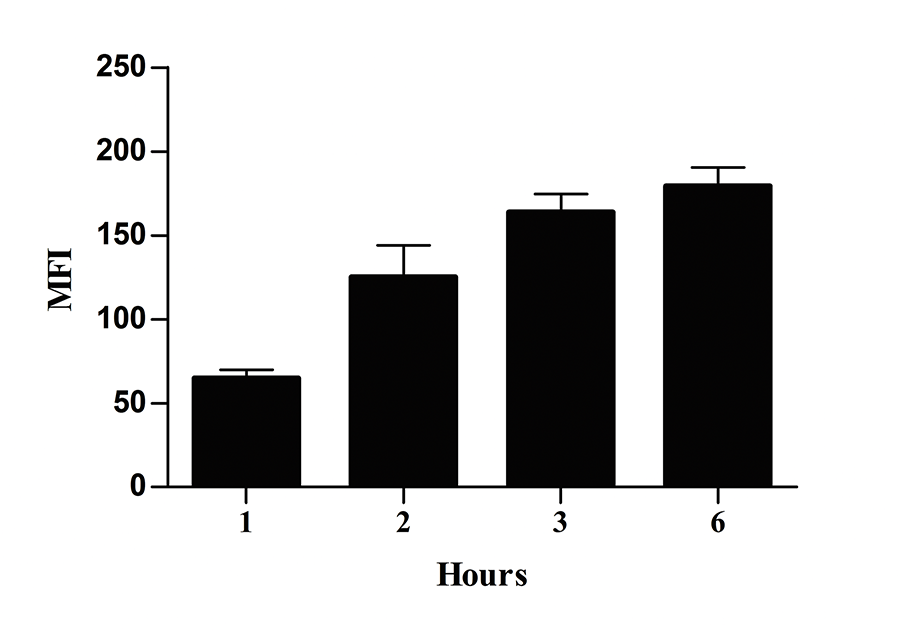

Supplement: S2 Fig — Late-stage iRBC were treated with 1 μM FITC-conjugated STAD-2 for 1, 2, 3, or 6 hours and subsequently stained with 2 μg/mL Hoechst 33342 before analysis by flow cytometry. Near-maximum levels of STAD-2 uptake are evident by 3 hours post-treatment (n = 3, mean ± S.E.). (TIF) [file pone.0129239.s002.tif]

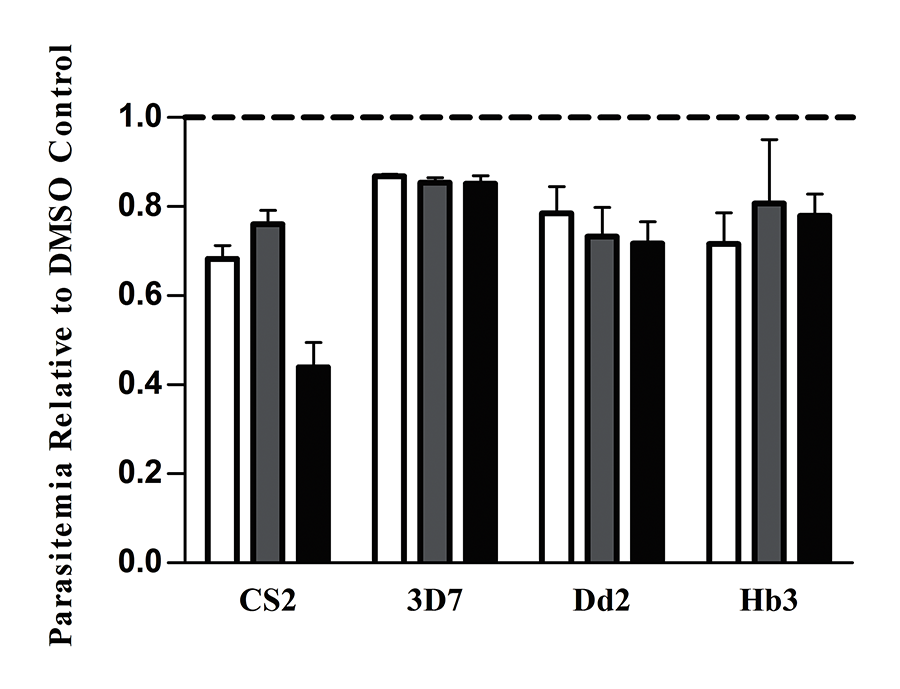

Supplement: S3 Fig — Late-stage CS2, 3D7, Dd2, and Hb3 parasite strains were treated with 1 μM FITC-conjugated STAD-2, and parasitemia was determined by flow cytometry at 24, 48 and 72 hours post-treatment. STAD-2 demonstrated variable antimalarial activity between strains and reduced viability in CS2, Dd2, and Hb3 strains (n = 3, mean ± S.E.). (TIF) [file pone.0129239.s003.tif]

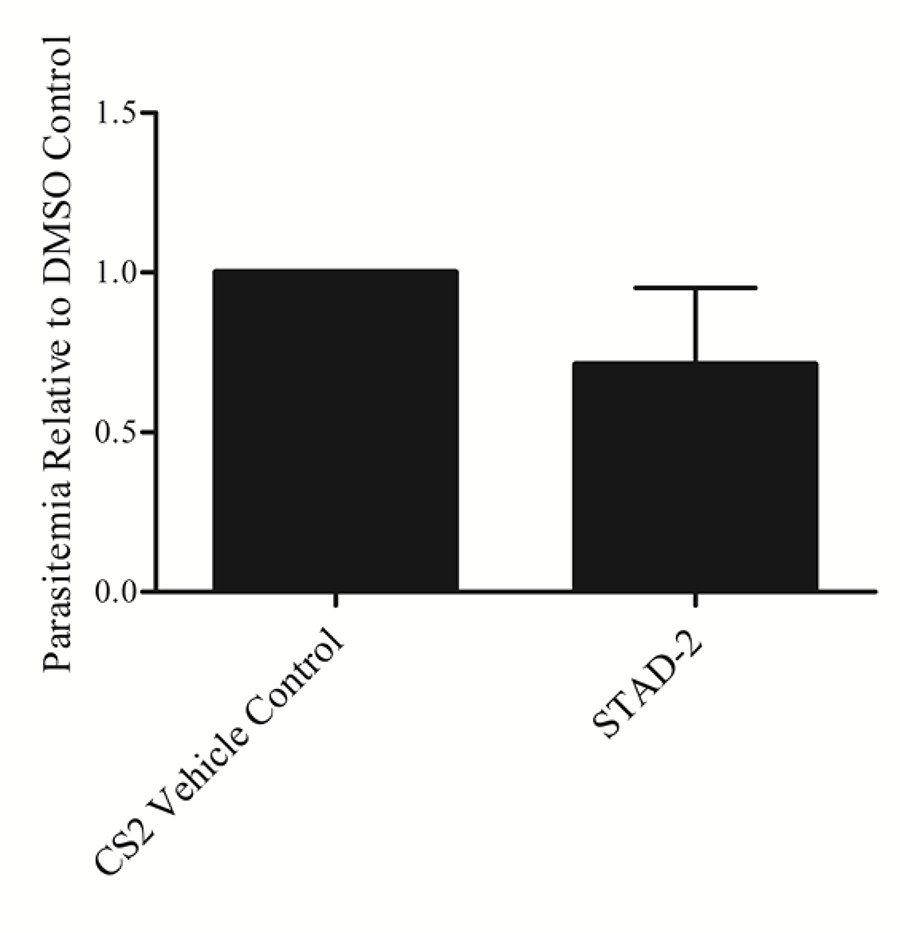

Supplement: S4 Fig — Late-stage iRBC were treated with 1 μM FITC-conjugated STAD-2 for 6 hours, stained with 2 μg/mL Hoechst 33342, and analyzed by flow cytometry. Reduction in parasitemia was evident as early as 6 hour post-treatment (n = 7, mean ± S.E.). (TIF) [file pone.0129239.s004.tif]

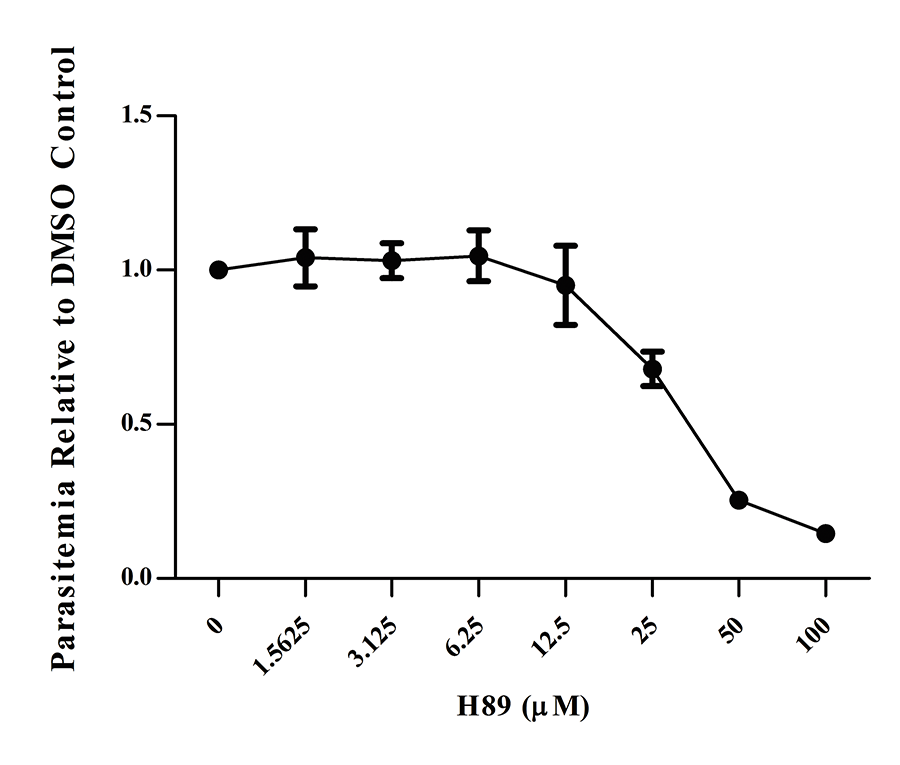

Supplement: S5 Fig — Late-stage iRBC were treated with serial dilutions (100, 50, 25, 12.5, 6.24, 3.13, 1.56, 0.78, and 0 μM) of the small molecule PKA inhibitor, H89, for 24 hours and stained with 2 μg/mL Hoechst 33342 for parasitemia analysis by flow cytometry. H89 in vitro IC50 ≈ 30 μM in late-stage CS2 parasites (n = 2, mean ± S.E.). (TIF) [file pone.0129239.s005.tif]

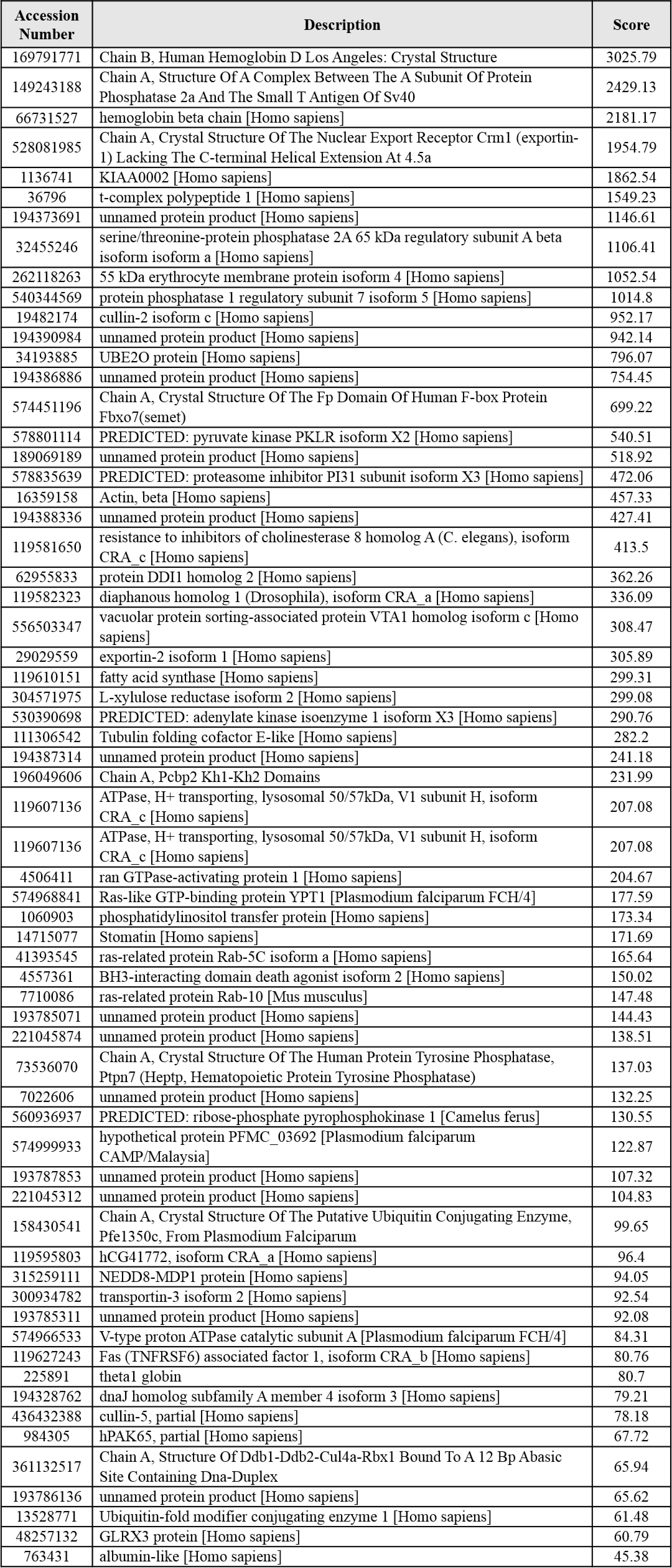

Supplement: S1 Table — Identified STAD-2 interactors from host supernatant following IP and LC-MS/MS. Interactors are ranked based upon their Mascot ions score. (TIF) [file pone.0129239.s006.tif]

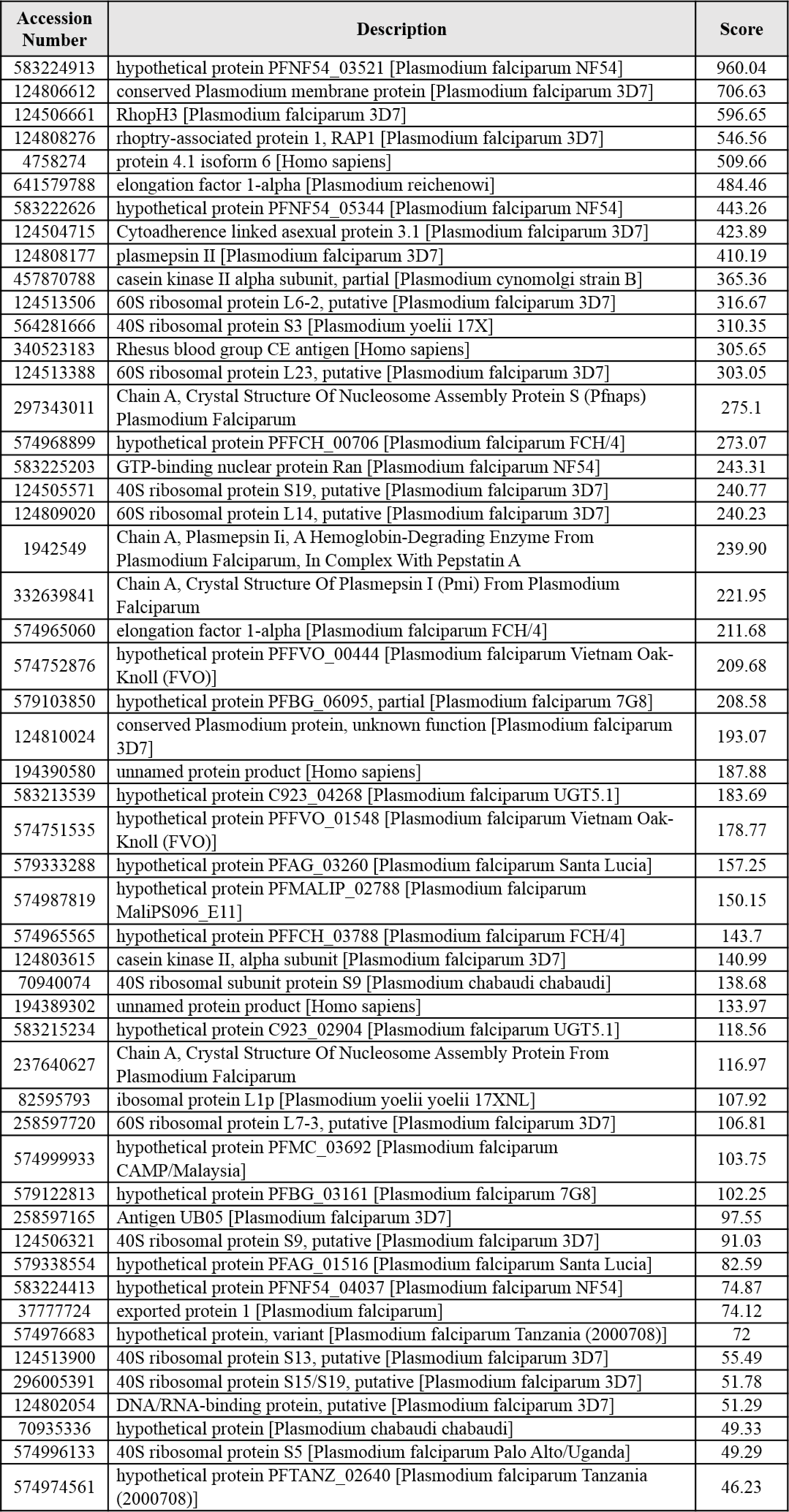

Supplement: S2 Table — Identified STAD-2 interactors from parasite supernatant following IP and LC-MS/MS. Interactors are ranked based upon their Mascot ions score. (TIF) [file pone.0129239.s007.tif]
